# A qualitative exploration of service users’ and staff members’ perspectives on the roles of inpatient settings in mental health recovery

**DOI:** 10.1186/s13033-020-00347-w

**Published:** 2020-03-06

**Authors:** Clara De Ruysscher, Stijn Vandevelde, Peter Tomlinson, Stijn Vanheule

**Affiliations:** 1 Department of Special Needs Education, Ghent University, Henri Dunantlaan 2, 9000 Ghent, Belgium; 2 Villa Voortman, Vogelenzangpark 10-17, 9000 Ghent, Belgium; 3 Department of Psychoanalysis and Clinical Consulting, Ghent University, Henri Dunantlaan 2, 9000 Ghent, Belgium

**Keywords:** Psychiatry, Recovery, Inpatient support, Complex mental health needs, Qualitative research

## Abstract

**Background:**

Today, international mental health care increasingly focuses on creating recovery-oriented systems of support. This study aims to unravel the daily practice of an inpatient psychiatric ward that engages with persons with complex mental health needs.

**Methods:**

17 in-depth interviews were conducted with patients and staff of the ward. Data was analyzed by means of thematic analysis.

**Results:**

Three important functions of the ward were identified in the participants’ experiences. First, it functions as an *asylum*, a safe environment where patients can ‘simply be’. Second, the ward is experienced as a *particularizing space*, as support is organized in an individualized way and patients are encouraged to reconnect with their own identity. Third, the ward functions as a *transitional space* towards a valuable community life, in which finding adequate housing is of central importance.

**Conclusions:**

The results show that inpatient forms of support tally with personal and social dimensions of recovery and fulfill important roles in recovery-oriented systems of support.

## Background

Under impetus of the deinstitutionalization wave from the 1960s onwards, international mental health care has been undergoing a reform in an effort to offer more adequate and holistic support to persons with mental health needs [1, 2]. In that reform, and especially over the last two decades, policy makers and practitioners have increasingly adopted the concept of recovery as a guiding principle, that is generally understood as a personal journey towards a meaningful community life, even with limitations caused by mental health problems [3–6]. Inclusive citizenship, feelings of connectedness and belonging, a positive social identity, meaningful activities (e.g. employment), self-determination and a sense of empowerment are considered key features of this process [7–9]. Influenced by these recovery ideas, the focus of international mental health care has shifted from a primarily medical approach to the creation of recovery-oriented systems of support that are anchored in the community and are characterized by person-centeredness and a flexible collaboration between different sectors (e.g. mental health care, addiction treatment, the forensic sector, community social work) [6]. In the Belgian context, for example, this shift towards recovery-oriented support has led to the reduction of beds in residential psychiatric care, a growing focus on short intervention-based treatment and the development of more support facilities in the community, such as mobile (crisis) teams and sheltered housing for persons with mental health problems [10]. At the same time, there is growing concern about this systemic transformation and the professional adoption and implementation of recovery-oriented principles in mental health care [11–13]. For example, authors warn that the concept of recovery risks being used as an incentive for funding and service cuts [11], risks being translated as an overly intrapersonal process in which the impact of social inequalities on mental health problems is overlooked [9, 13], and risks turning into an expert-driven discourse in which deficit-oriented language is still latently present [14], amongst other things.

Whilst residential treatment in psychiatric hospitals still makes up a large part of today’s mental health care, the implementation and operationalization of the recovery concept has primarily and most explicitly taken place in outpatient and community settings [15]. Many go further and assert that implementing the principles of recovery-oriented practice in more traditional inpatient settings is an extremely challenging task [2, 16]. For example, a recent study of Waldemar and colleagues shows how recovery-oriented values (e.g. a focus on patients’ personal preferences) in such settings often remain overshadowed by competing priorities (e.g. complying to ward rules, power imbalances between staff and patients) and organizational hospital logics (e.g. bed capacity) [17]. However, there remains a substantial group of persons with complex and long-term mental health needs for whom more lengthy and intensive admissions to psychiatric hospitals continue to be a necessary part of their recovery journey [18, 19]. Whilst this is a heterogeneous group, the complexity of their support needs can often be situated at the intersection of the mental health problems they face and multiple other problems such as homelessness, substance abuse, poor physical health, poverty and judicial problems, often leading to experiences of exclusion, long treatment trajectories and social isolation [20, 21]. As a result, the recovery processes of persons with complex mental health needs have a slow and unpredictable course and are characterized by many ups and downs [22]. Although admissions to psychiatric wards play a significant role in their recovery process, most of the scientific evidence on recovery-oriented practice has been developed in organizations that are located outside the hospital walls (e.g. community-based initiatives, outreaching support and case management) [4].

If we want to avoid having persons with complex mental health slipping through the net of recovery-oriented systems of support, it is crucial to understand *how* these inpatient spaces play a role in their recovery journeys. Considering the idiosyncrasy of recovery processes and the versatility of such settings, several authors have recommended the use of qualitative research approaches that are context-close, focus on micro-level recovery dynamics and are grounded in the lived experiences and perspectives of the persons who are directly involved in the practice under study [7, 23–25]. Therefore, the aim of this study is to gain insight into the different functions these inpatient settings have in the recovery processes of persons with complex mental health needs, by unraveling the daily practice of one ward (located in a psychiatric hospital in Belgium) that actively engages with this complex and long-term patient population. More specifically, this study aims to study the daily practice of the ward by focusing on the personal perspectives and lived experiences of different involved actors (i.e. staff members and patients).

## Methods

### Methodological approach

This study aims to gain insight into the daily practice of the ward by focusing on the patients’ and staff members’ personal experiences. Therefore, a qualitative research approach was applied [26]. Data were collected by means of in-depth interviews and analyzed using thematic analysis [27].

### Research location and participants

The study was carried out in a residential ward of a large psychiatric hospital in Flanders (the Dutch-speaking part of Belgium), that offers support to a diverse group of patients with serious and more chronic mental health problems, who have often lost connection with other treatment settings. Besides mental health problems, many patients on the ward deal with substance abuse problems, a lack of social network, financial and judicial problems, and housing problems (more than 50% of patients on the ward are homeless). Rather than focusing on treatment, the ward aims to support patients in their recovery process towards a meaningful life in society. Considering the heterogeneity of the patient group, the ward offers several support modalities that are used in an individualized way, depending on the personal needs and situation of each patient. The largest part of the ward consists of a residential unit with 27 beds. Additionally, the ward runs a housing project (attached to the hospital) in which six patients live together more independently (for a period of maximum 1 year) after a stay on the residential unit, in preparation for living alone. Besides residential support, the ward also offers aftercare for patients when they leave the ward and outreach case management for patients who find it difficult to engage with a residential treatment approach.

To obtain rich and triangulated perspectives on the daily practice of the ward, both patients [10] and staff members [10] were recruited as participants for in-depth interviews. Patients were deliberately selected based on their treatment history and the way in which they make use of the ward (e.g. residential unit, housing project, case management). The patient participant group consisted of seven men and three women, aged between 23 and 59. At the time of the interviews, two patients were staying at the housing project, six patients were staying at the residential unit (two of which were interviewed together) and two patients visited the ward a few times per week for aftercare. The participants’ length of stay on the ward varied greatly (ranging from weekly outpatient visits to inpatient admissions of several years), and most participants had already gone through several periods of admission at the ward for shorter (e.g. a number of days/weeks) and longer (e.g. a number of years) terms prior to the moment of the interview, depending on their recovery process. The staff sample consisted of three men and seven women, aged between 25 and 58. In the recruitment of the staff members, we aimed at obtaining a wide diversity of professional backgrounds. The staff participants consisted of a psychiatrist, two psychologists, a social worker, two psychiatric nurses, a pedagogical staff member and three occupational therapists (who were interviewed as a trio). All 20 participants were considered to be suitable informants, based on their lived or professional experience with the ward.

### Data collection and analysis

To prepare for the interviewing phase, the first author did an internship on the ward from April to June 2018. During this internship, she enrolled in the staff shift system, took part in therapeutic group activities (such as the daily morning meeting), staff meetings and individual activities with patients, and engaged in many informal conversations with both staff and patients. In doing so, she gained a better understanding of the daily practice and became familiar with the staff and patients on the ward. Additionally, the internship facilitated the recruitment of participants and the first author was able to be more sensitive to the emotional well-being and motivation of patients in planning and conducting the in-depth interviews. Also, the internship allowed her to make an adequate estimation of the diversity in the staff and patient population and to represent this diversity in the participant sample. A total of 17 in-depth interviews were conducted (some participants preferred to be interviewed as a duo or a trio) that focused on the participants’ personal perspectives on the daily practice of the ward. During the interviews, participants were asked about important values and activities on the ward; about the function(s) the ward fulfills in patients’ lives and society; and about social dynamics on the ward. Interviews lasted between 28 and 68 min, were audio-recorded and transcribed verbatim by the first author.

In the analysis phase, the first author conducted an idiographic analysis of each interview by reading the transcript several times, writing down descriptive and interpretive comments, and drawing a mind map of emerging (dynamics between) themes. In this initial stage of data analysis, the insider perspective that the first author had gained during her internship on the ward served as helpful background information to contextualize, interpret and structure the emerging findings. After this initial analysis of each interview, mind maps were gradually combined to gain insight into overarching themes and into the ways they interrelate, complement or are in tension with each other. In the first step of this integration phase, the first author selected five key interviews based on their thematic richness and the diversity of perspectives. These interviews were independently analyzed by all co-authors and integrated into one mind map, which was extensively discussed. Based on this discussion, the first author completed the entire analysis, which resulted in an overall thematic structure. In turn, this structure was discussed again in a meeting with all co-authors. Through this iterative process, the authors aimed to increase the inter-rater reliability and to deal with the subjective nature of the analysis.

Although this article is written from a social science (rather than a medical) perspective, we will continue to use the term ‘patient’ (rather than ‘client’ or ‘service user’), as this terminology was used by the participants during the study.

### Ethical considerations

This study was granted ethical approval by the Ethics Committee of Ghent University Hospital (EC UZG 2016/0530). Written informed consents were obtained from all participants.

## Results

The aim of this study is to unravel the different roles the ward plays in the recovery processes of its patients. In that respect, three main functions come to the fore in the experiences of the participants. In the first place, the ward fulfills the function of an *asylum*, i.e. a safe environment where patients feel sheltered, allowing them to catch a breath and ‘simply be’. Within this safe environment, the patients’ psychiatric diagnosis does not determine their therapeutic trajectory. Instead, through offering an individualized and tailor-made approach, patients are actively encouraged to take (back) agency over their recovery process and to (re)connect with their personal identity. From this follows the second function of the ward: that of a *particularizing space*. Importantly, the focus of this individualized approach is always on finding and creating anchor points in the community, rather than on functioning within the safe shelter of the ward. This leads to the ward’s third function: that of a *transitional space* towards a valuable life in the community. Providing as much continuity between life outside and inside the ward as possible and searching for adequate housing are key elements of this function.

### The ward as an asylum

All interviewed patients talked about how they see the ward as a safe haven. In the experiences of the participants, three aspects of this asylum function were distinguished: feeling safe, feeling ‘at home’, and the ward as a safety net. In what follows, these will be discussed in detail.

#### Feeling safe

Most patients arrive at the ward after a turbulent period of being homeless, spending time in prison, facing difficulties living alone (e.g. social isolation, mental health problems, substance use) or after a long trajectory in other (more treatment-focused) psychiatric settings. For many of them, a primary function of the ward is that of a safe haven and a place to catch a breath. In the first place, the fact that a number of basic needs, such as having access to food, a bed, laundry facilities and a daily structure, are met during their stay is of fundamental importance, as these things are often not self-evident in the patients’ lives. At the same time, patients are not pressured into taking part in a strict therapeutic program. As a result, they feel like they are given comfort, time and space to rest and recover from the turbulent period they went through. Because everything is voluntary, patients themselves are in control of the pace of their stay, which adds to their feeling of safety:

*“It is important for me that when I feel down or sleepy, that I can take a rest here and that nobody is shaking my bed. Sometimes they check on me, but that I shouldn’t feel embarrassed when I really have an off*-*day. That I can be myself in that too.”* (patient staying on the ward, male)

In addition, for some patients, the constant presence and close proximity of the staff on the ward is another invaluable condition to be able to feel safe and at ease:

*“At home I always sit inside, me, at home. But here I go for a walk every now and then. (…) Here I find more peace than at home, I must admit. There are nurses day and night, that reassures me. In sheltered housing, if I don’t feel well after four pm, nobody’s there. But here I can count on a nurse. That reassures me…”* (patient, male, staying on the ward)

In other words, the proximity of the staff and the presence of all basic services provides patients with a sense of security. At the same time, however, using the ward as a safe haven also brings the risk for patients getting so accustomed to these comfortable circumstances that they become less inclined to pick up their lives outside the ward, where the fulfillment of basic needs such as having food and shelter are less self-evident. This risk is also pointed out by one of the patients:

*“You feel safe. You have food every day. In the beginning that is good, because you feel bad. But after a while you feel like ‘oh, I’d like to cook myself again’. Or ‘how on earth am I going to do this again at home, making food again?’”* (aftercare patient, female)

#### Feeling ‘at home’

Some patients on the ward have been living at the psychiatric hospital for more than 10 years. These long-term admissions flow from an outdated institutional reasoning that for some patients, life outside the hospital is too challenging and that the hospital can function as their life-long safe haven. Having lost contact with their social network and social roles outside the hospital, these patients experience the ward as their home. However, this is an unintended function, as the ward primarily aims to help patients find a meaningful place outside the hospital. This shows how the ward’s current shift from an institutional logic to a more recovery-oriented logic confronts these long-stay patients with a new set of expectations (i.e. focusing on a life outside the hospital rather than feeling ‘at home’ inside). In that respect, one of the psychologists explains how challenging it can be to create a perspective on a life outside with this group of long-stay patients:

*“There are people who’ve lived here for more than ten years. (…) In such cases it is difficult to say that people don’t feel at home here. People do. At the same time, in the same sentence, we need to add that we should also keep trying to also give those people some kind of perspective outside the walls of the psychiatric center. (…) But it has to be a perspective that is acceptable to them and in which they feel that they have some ownership, some choice.”* (psychologist, male)

According to ward’s psychiatrist, however, it can be particularly difficult to bring out this agency and sense of ownership in patients who have been at the ward for years, as they have grown so accustomed to life inside the hospital that they can no longer imagine feeling at home anywhere else. She refers to this danger as the ‘hospitalization syndrome’:

*“People were used to the idea that they could live here. They were even told by the staff that they could stay here for the rest of their lives. (…) But you just know they can’t imagine how it is like to make yourself a cup of coffee when you wish, or to have your own room. They just don’t remember what it is like to have privacy and to… So actually they need to experience it first hand before they… Before they can let go of a place like this and move on to a more uncertain… So sometimes, it doesn’t work out.”* (psychiatrist, female)

The ward aims to counteract the effects of this syndrome and to keep a perspective on the outside world alive, by actively encouraging patients to find activities in the community, help them (re-)build a social network and support them in finding adequate housing. However, dispirited by long and often unsuccessful previous treatment trajectories, some patients consider these efforts pointless. One of the patients explained how he finds the asylum function of the ward the most important at this stage in his life:

*“For some people it should be comfortable. I am not saying that for people who just start their career, to say it like that, who have their first admissions, it should be like that. But for people who have been through a lot, you shouldn’t make too much effort. Leave people be, and give something, a place where people can feel good. That’s all it should be.”* (patient staying in the housing unit, male)

#### The ward as a pit stop

After discharge, most patients return to the ward on a regular basis for a few months, as aftercare. For most patients this means spending 1 or 2 days a week at the ward, taking part in activities and seeing the psychologist. Other (ex-)patients continue to use the ward more intensively by returning regularly for shorter admissions (e.g. 1 or 2 weeks). Talking about how she found balance in her life after having spent long periods in the psychiatric hospital over the past 24 years, one of the aftercare patients explains the importance of these short returns to the ward:

*“I think I’m happy with the balance that I’ve found. (…) The periods that I spend a little while here, I have to let go of everything. My volunteering job, my household. (…) It’s a few days of not thinking of anything and just being here and doing things that I love doing, which I don’t have time for at home.”* (aftercare patient, female)

In this way, the asylum function of the ward carries on after discharge, as it remains a pit stop for ex-patients after they have left. Although the aftercare phase is an essential part of the therapeutic trajectory, its aim is to be as short as possible, as ex-patients increasingly find more meaningful activities in their community. However, this is in contrast with the way some external partners ‘make use’ of this aftercare program. For example, for sheltered housing organizations, having structural daytime activities (e.g. a paid job, volunteering, a hobby) is a prerequisite to get a place at a housing unit. When (ex-)patients have not found such activities yet, the aftercare program is often considered as a ‘good enough’ structural daytime activity by these organizations. Consequently, (ex-)patients are under pressure to keep coming to the ward instead of engaging in other more meaningful activities in the community. In other words, the logic of these organizations sometimes contradicts the ward’s recovery-oriented way of working.

To counteract these dynamics, the ward actively provides support and coaching to both professional (e.g. mobile teams, estate agents, judicial partners, social housing organizations) and informal (e.g. family, neighbors) partners that are part of the support networks of their patients. In that sense, the ward fulfills a function as a pit stop not only for its patients, but also for these organizations and partners. One of the staff members describes how providing such a safety net opens up new spaces for external partners to engage with (ex-)patients, as they have the insurance that the ward will stand beside them and guarantee adequate support in crisis situations:

*“What I am often confronted with… When you try something with someone, that you almost guarantee that, if it would go wrong for the client, that you offer the service that partners aren’t left alone with it. (…) Often, partners have many questions, but are also looking for reassurance: okay, we want to work with that patient, but what if it goes wrong? Instead of leaving that person to his fate, can he come back to you? (…) And I notice, if you can offer that guarantee, people dare to take more space to try something.”* (pedagogical staff member, female)

### The ward as a particularizing space

The asylum function of the ward creates a safe climate, in which it becomes possible to offer support in a tailor-made and individualized way. In that respect, the patient’s therapeutic trajectory is subject to a continuous process of negotiation between patient and staff. Through such a particularizing approach, space is created for patients to (re)connect with their personal identity and aspirations. In this section, these aspects will be elaborated.

#### A tailor-made approach

The patient population at the ward is characterized by a large diversity in terms of mental health problems, biography and the shape their recovery process takes. Consequently, all patients have different motives for spending time at the ward, as one psychiatric nurse describes:

*“For example, Tom finds it really annoying to be here, it is like a love*-*hate relationship. He is polite and he knows he sometimes needs it, but he is… Yeah, at the same time he hates it, being here. He finds it awful. Then… Do you know Leanne? (…) A very compulsive neurotic woman who also has psychotic outbursts every now and then. She lives alone, but has spent a long time here, and sometimes asks for a (short) admission. She can clearly define that for herself, ‘I will stay here this long and then I can carry on again’. (…) And I think we have a bit of everything in between [these extremes], a bit between Tom and Leanne.”* (psychiatric nurse, male)

Given the great heterogeneity in the motives and support needs of the patients, it would be inadequate to provide a one-size-fits-all therapeutic group program. Instead, the ward aims to give shape to an approach that is tailored to the recovery process of each patient. Rather than starting from a predefined therapeutic program that comes with a set of expectations (‘what do we want from the patient?’), patients are encouraged to formulate what they expect from their admission and how it can support their recovery process. In doing so, they are actively invited to give meaning to their stay on the ward themselves, thus take (back) agency over their lives:

*“Here, everything is very open. A lot more like, yeah… You (the patient) tell us what to do. And that’s what we work with. We are not going to tell you here how you should do it, but you have to do it for yourself.”* (occupational therapist, female)

*“Having a low threshold. Not setting too many conditions for someone to come to the ward. (…) Actually, you put forward that the aim is to travel a road that is very individual. (…)What does one expect of one’s future? How does one picture that? What does one need? And we try to anticipate that. (…) But we try to do it in a very individual way, because it should always fit like a key in a lock.”* (psychologist, female)

This particularizing approach is also reflected in the ward’s policy regarding substance use. Although using alcohol or drugs on the ward is not allowed, there is also no zero-tolerance policy. Instead, an individual approach is outlined for each patient, that corresponds to that patient’s personal needs and wishes. In other words, in the ward’s vision on recovery, abstinence is not put forward as a fundamental precondition. One of the psychologists explains how such an individual approach can possibly take shape:

*“If someone says ‘I want to use cocaine weekly for the rest of my life, but I can do it in a limited way and I can do it once a week on Saturday night at a friend’s house’, then we won’t… As long as his cocaine use doesn’t stand in the way of his trajectory towards outside… If he has enough money to rent a house or something else, then we won’t make a problem of it. (…) But imagine that the same man says ‘I want to live in sheltered housing, anything else isn’t possible for me’, then we will make a problem of it, because no sheltered housing unit will allow [people who are not abstinent].”* (psychologist, male)

In other words, the ward’s substance use policy is attuned to the recovery process of each patient, as it is the result of a negotiation between staff and the patient. Consequently, patients gain a sense of ownership over these rules. For example, one patient talks about the nuances in the reactions of the staff regarding the use of drugs depending on the patient involved:

*“They approach everybody individually here. And I think that they know of some people that they drink something every now and then, or smoke a joint, and that they turn a blind eye. Well, that’s what I think. And they can’t do that for me, because it is not allowed. If they know that I drink, they are legally obliged to pass it on.”* (patient staying on the ward, male)

#### Reconnecting with one’s identity

In their daily lives, patients are often confronted with stigmatizing experiences in which their identity is narrowed down to their psychiatric label or their status as a homeless person. Facilitated by the individualized approach of the ward, however, patients are not reduced to their psychiatric diagnosis but are rather seen as individuals with unique aspirations and vulnerabilities. One of the psychologists explains how the staff on the ward actively tries to oppose a narrow interpretation of the patients’ identities:

*“Definitely when you’re homeless, you don’t have many friends or acquaintances, you don’t own things. And then you’re put into a system where the only thing that is left of your identity is that you are a patient. And that can… work in a paralyzing way, depressing way, make you apathetic if you stay in it. So it is an art to listen… like what do you identify yourself with that might be snowed under by your patient identity?”* (psychologist, male)

By actively engaging in dialogue with patients about their personal interests and social roles, a new dynamic is created in which they are challenged to (re)connect with and show these aspects of their identity, thus shifting their ‘illness identity’ to the sideline. Consequently, patients are not approached as passive recipients of a certain treatment but rather as active agents of their own (personal and social) recovery process. In this respect, one of the patients points out how this activating approach caused a shift in his mindset regarding his own recovery process:

*“There (at another ward) I had the impression that they had a prejudice or an image that something was wrong with me, that I had symptoms, those moments of illness or when it became acute… Something that had something to do with my mental problems. And that it is disabling. And here, indeed, it is more… I grow conscious of the fact that it is just… That it is about functioning… How can I function outside the admission?”* (patient staying on the ward, male)

This shows how, through widening the lens on their identity, patients are no longer seen (and see themselves) as fundamentally different, i.e. as mentally ill persons who need to recover clinically. Rather, they are approached as individuals who try to give meaning to their lives in fundamentally similar (personal and social) ways to most people. This is reflected, for example, in the experiences of one of the patients who stresses the importance of being able to fulfill valuable social roles in the community:

*“First I need to find some day activities. Maybe a volunteering job, then that would also be fine. But I am going to work so that I can find my own apartment, my own job. I just want to get away, I want to get back into the system, work every day so I have money in my pocket. So that people look at me respectfully because I work.”* (patient staying at the housing unit, male)

In its daily practice, the ward strives to support patients in establishing a personal and social identity. As this support is powered by the patient’s own interests and aspirations, it can take on many forms, such as searching for meaningful activities in the community, restoring contact with family, finding a place to live, helping applying for volunteering/paid work, developing an interest or skill, and so on. Paradoxically, helping patients (re)connect with their personal identity is at times hindered by factors that are characteristic of the culture of the psychiatric hospital itself. For example, the psychiatric hospital has to comply with legislation in which a set of rules and regulations regarding the hygienic and safety standards of the provided care is specified. However, one of the psychologists gives a list of illustrations of how these imposed rules can stand in the way of a more tailor-made and identity-focused approach to support:

*“Often it is about really practical things, such as… letting patients decide about certain things. It is about food, about TV, about… (…) You can’t let family stay over, because of the fact they’re all hospital beds. People who have a pet, who need to find shelter for it because it is a hospital. But also really small stuff… Having a piano on the ward, because of fire safety. Such things. Having your own key to your room. I could spend a whole day listing these things.”* (psychologist, male)

Also at the level of the ward itself, different factors (e.g. the large patient population, the absence of a mandatory treatment program, the large amount of meetings, having an individualized rather than a group-oriented approach) can at times make the atmosphere on the ward hectic, even chaotic, leading to more superficial and fragmented conversations between staff and patients and leaving less room for quality interactions such as one-to-one trips outside the hospital.

#### A continuous (re)negotiation

An important implication of offering an individualized approach to support on the ward is that patients do not follow a pre-structured, one-size-fits-all therapeutic group program. As patients are encouraged to take agency over their own recovery process, support takes the shape of a continuous (re)negotiation, both between the staff and each patient and between the staff members themselves. Consequently, the therapeutic trajectories that patients travel during their stay often take a less straightforward, less predictable course. One of the psychiatric nurses explains how this requires a particular attitude from the staff, that is different from other more traditional inpatient settings:

*“I have worked at a ward with more rules, and that is very comfortable but also quite blunting, because you… You are just a representative of rules that you can hide behind. And here, that is way less the case. It is a tiring reflex to continuously find the particular… In consultation with the patient or with each other, to get to a logical plan of action. It is more intensive, but I believe in it. (…) At the same time I hope it is that vision, that you can’t simply summarize people or practices into something that is easy to capture. It is all very difficult to capture and that takes time and communication and re*-*inventing.”* (psychiatric nurse, male)

In such a process of negotiation, staff members take the position of travel companion rather than guide in the recovery journey of the patients, as one caregiver explains:

*“That’s recovery. That you never say to someone… In the beginning, this is not going to work. But that you stumble over the same things together and that you keep standing beside that person and that you try something different, something that person can identify him/herself with.”* (pedagogical staff member, female)

In doing so, patients are given as much ownership as possible over their own trajectory, but this can also bring new difficulties. One staff member explains how she finds it difficult to take the position of travel companion when the direction the patient chooses is, in her opinion, not realistic:

*“But what I personally find difficult sometimes, is when you have the feeling that what someone wants is very unrealistic. (…) But that is also part of the job of course. (…) From that perspective I can frame it and I know it is linked to recovery, with ups and downs, and that people have to learn to discover things for themselves. Or rather, that we need to let them discover.”* (social worker, female)

In other words, offering particularizing support is time-intensive and requires space for trying different things, starting over and thinking outside the box of traditional options. However, in the daily practice of the ward, precisely this improvisational space is continuously under pressure from macro-level (e.g. government, hospital) policy decisions regarding personnel and finances. For example, to meet the patients’ individual needs, the ward offers a wide range of support modalities (e.g. case management, outreaching support, a housing project), which is a very challenging task compared to the financial resources of the ward. Also, during weekends the ward is only staffed by one or two people. One of the psychiatric nurses expresses how he finds it frustrating to work at weekends, when the ward can only fulfill its asylum function because of staff shortage:

*“When you are alone here, you are like a walking key who just keeps things going a bit. Then you can’t really speak of rehabilitation. (…) The only people who are here then, are the ones who are bored, who don’t have much outside, who aren’t always feeling very well. Then I rarely go home happy.”* (psychiatric nurse, male)

This example illustrates how the economic logic of the hospital (i.e. the financing and allocation of personnel) can sometimes be in conflict with the particularizing vision of the ward.

### The ward as a transitional space

The third function of the ward that can be distinguished in the experiences of the participants is that of a place of transition towards a meaningful community life. When discussing this function, two themes come to the fore: minimizing disruptions between life inside and outside the ward and finding adequate housing.

#### Continuity between inside and outside

Many patients on the ward have experienced previous admissions as major disruptions to their everyday life. The ward aims to avoid such disruptions, by striving for a seamless continuity between life ‘inside’ (i.e. spending time on the ward) and ‘outside’ (i.e. having a valuable community life), both by bringing in the outside world (e.g. by talking about it) and by actively supporting patients in finding a meaningful place in the community (e.g. by searching for a place to live, restoring contact with family, searching for hobbies). Considering the heterogeneity of the patient population, the psychiatrist describes how challenging it can be to work towards a life outside with/for the patients on their ward:

*“Here, we really need to work hard towards outside. (…) Because actually you still need to create the outside. You still have to register them on a waiting list for sheltered living or the social housing company. And sometimes the work will never be finished, because they will never make it outside. Or working outside means installing the idea of outside, that there is something else besides the ward.”* (psychiatrist, female)

One of the psychologists points out how the psychiatric institution in itself has an alienating effect on the patients’ everyday lives. More precisely, he warns for the risk of installing a false dichotomy of the ward as a safe shell versus the outside world as threatening. From that perspective, he argues that a continuous focus on the outside world in the ward’s daily practice offers a vital complement to the asylum function of the ward:

*“It always surprises me when people are told “you’re safe here, outside it is dangerous”. (…) To say it in psychoanalytic terms, you’re working in a very dual way, I find. (…)So it comes down to saying “you are here and I am here and outside we might find some people who could be interesting and reliable, I don’t know”. (…) It is by talking about the other that you install a third point.”* (psychologist, male)

Another essential facilitator of this continuity between life inside and outside the ward is to install a non-controlling climate, in which patients are not punished for their setbacks or mistakes but remain welcome on the ward and are encouraged time and time again to pick up the thread of their lives. Talking about this, one of the caregivers stresses the importance of applying a long-term vision to the recovery trajectories of the patients they work with, to prevent bridges being burnt:

*“We work with long trajectories. To continuously try again. For example, someone who’s having a hard time on the ward, to give him a break, such as asking for a time*-*out, but to then… always give him or her the chance to return if that person wants that. At the moment, I don’t think that is the case on other wards. (…) And I notice that people find it easier to return to us too. That it is an interruption, but not a stepping out of the trajectory.”* (pedagogical staff member, female)

This climate also becomes visible in the fact that patients are given as much freedom as possible by not obliging them to take part in the available therapeutic activities (e.g. cooking therapy, creative therapy, music therapy, sports activities). As a result, they are more inclined to engage in meaningful activities outside the ward. For example, one of the patients explains how the fact that no obligated therapeutic program is imposed on him motivates him to work on his living conditions outside the ward:

*“I like it here, that the activities are not mandatory. If they would be mandatory, yeah, then I’d… I’d take part in them, but there wouldn’t be much benefit for me. (…) Actually, now I mainly need to do things outside the clinic, like cleaning up my apartment and stuff. Yeah, and if I can do sports here, or reading together or… Then that is nice, but I mean, in the first place I need to do some things outside if I want to feel better.”* (patient staying on the ward, male)

The above experiences clearly show how the ward’s mission to provide continuity between being admitted and living in the community also entails a shift in the job description of the staff working on the ward, from a traditional institutional approach towards a more community-oriented way of working. This shift is formalized in the job description of certain staff members. For example, three staff members practice the function of ‘road builders’ (‘spoorleggers’) and actively search and create spaces (e.g. libraries, sports centers, voluntary/paid jobs, cultural events) in the community that welcome people with mental health problems. In doing so, they actively pave the way (hence the name ‘road builders’) for the patients on the ward. In that sense, their primary function is that of ‘quarter making’ (‘kwartiermaken’), i.e. actively creating hospitable niches in society for people with mental health problems, who are often confronted with exclusion and stigma [28]. Another group of staff members work as case managers for patients who have difficulty engaging in the ward’s residential unit for a long period of time, and continuously move in and out of the ward. As a result, not only in the patients’ experiences, but also for the staff, the boundaries between inside and outside become more blurred and porous:

*“There is some sport, and music therapy and stuff, but most of the therapists are already looking towards the outside, or even the other way around. They are sitting outside and looking in and asking people ‘do you wanna come? There is an event in the library, we are helping with that, do you want to help too?’ That vibe. Yeah, that’s something I believe in strongly.”* (psychiatric nurse, male)

#### Housing first

Housing came to the fore as a prominent theme in all interviews. Since the majority of the patients on the ward are or become homeless during their admission, finding adequate housing forms an essential part of the patient’s transition process towards a meaningful community life. For example, one patient expressed how, for him, finding a place to live in which he can feel safe and at ease is a primary need:

*(interviewer) “When will you be ready to leave the ward, do you think?” (patient) “That is related to finding alternative ways of living that for me… So somehow I expect a search to find another place I believe in. (…) If I get discharged whilst I don’t believe there is a connection, I will go back into crisis. Period. This is no emotional blackmailing, that’s just how it goes. Then I go into destruction.”* (patient staying on the ward, male)

Although searching for adequate housing is a key function of the ward, the rhythm of this search is largely determined by external organizations and factors. For example, social housing companies, organizations for sheltered living and psychiatric nursing homes often have long waiting lists. Also, in some cases, it is not the patient but a judicial actor who decides if and when one is capable of living independently. Another factor that often impedes the search for appropriate housing, especially on the regular housing market, is the stigma that many patients have to face on a daily basis. One of the psychiatric nurses gives a striking example of the impact of this stigma on a patient’s search for an apartment:

*“I think we do miss opportunities sometimes, but… I can also see why. Jimmy (one of the patients) is my referee. I find it difficult to keep it going with him. Sometimes we go look at a house, and then he makes a bit of a strange impression. Maybe this will stick to the estate agent, and then we are back at… I would like to spend an entire day convincing landlords that actually, Jimmy is the perfect tenant. (…) He’s on benefits, that is pretty stable, I mean, that will keep going… And he is clean, he is very predictable. He is supported by us in case something goes wrong. So actually he is a catch, but I’d like to see that happen.”* (psychiatric nurse, male)

As a result of these external factors, patients and staff do not have control over the search for adequate housing, which has implications for the daily practice of the ward. According to one of the psychologists, the rhythm of the (regular/social/care) housing market is predominant in such a way that it makes it impossible to see the ward as a space where patients ‘are being prepared’ for a more independent life in the community. Instead, both staff and patients need to respond ad hoc to housing opportunities when they present themselves:

*“Deciding whether someone is ready, is something we actually never do. It depends on what is available at that moment. (…) Often, when a [housing] situation presents itself and the patient agrees, then we try to look which supportive framework we need to install, so that it might succeed. Does that mean that people are ready or not? Actually that is a question we never ask ourselves.”* (psychologist, male)

As a consequence of the fickle rhythm of the housing market, patients are more often discharged from the ward based on the availability of a housing spot than based on their ‘readiness’ to go. Although the staff always attempts to build a supportive network to make the move to the new living situation as smooth as possible, it does not always end successfully. Some patients need to return to the ward several times and make repeated attempts at living independently before finally succeeding in finding a stable place in the community. For other patients, the ward has become more of a ‘waiting room’, as it can take a long time until a housing spot is available:

*(patient staying on the ward, female) “Our problems are solved, so actually we could go, but yeah… We can’t go because we don’t have that one thing.” (interviewer) Because you don’t have a roof over your head? (patient) “Yeah.”*

## Discussion

This study aimed to gain insight into the different functions that inpatient psychiatric settings have in the recovery processes of persons with complex mental health needs. We investigated this by unraveling the daily practice of one ward that actively engages with this population. The results showed how the ward’s daily practice takes shape in the continuous dialectic between the inside and the outside world, in the tension between different (e.g. economic, therapeutic) dynamics, and in the—at times dissonant—harmonies between the rhythms of different actors involved (e.g. patient, ward, hospital, society). Within this entanglement, three functions of the ward could be distinguished: the ward as an *asylum* (i.e. a safe haven where patients are given time and space to ‘simply be’), the ward as a *particularizing space* (i.e. a place to reconnect with one’s identity and aspirations), and the ward as a *transitional space* (i.e. a place to work towards a meaningful community life).

Several aspects of these functions tally well with the personal, social and relational ways in which recovery is conceptualized in the literature [7, 9]. First, by applying a tailor-made approach to support in which patients are encouraged to explicate their expectations regarding their stay, the ward manages to connect with and provide an answer to the nonlinearity and idiosyncrasy of the patients’ recovery journeys [29]. Importantly, in this particularizing approach, personal recovery is not considered a solely intrapersonal process of gaining a sense of self-direction, developing resilience and independence, feeling hopeful about the future and establishing a sense of identity. Instead, at the ward, support takes the shape of a continuous negotiation in which both staff and patients stand side by side in searching for the most adequate response to the patient’s needs and wishes. This shows how recovery is above all a relational process that comes about in the interdependence between an individual, his/her social context and relationships (e.g. professional actors, personal and social network) [9, 16]. Additionally, the ward aims to keep this interdependence intact by minimizing disruptions between life inside and outside the hospital, and by developing a tolerant climate in which patients are not obliged to follow a set therapeutic program (allowing them to spend time outside the ward) and are not punished for setbacks (that can often be related to the fickle nature of their recovery). However, due to experiences of stigma and exclusion, these positive social contexts and relationships are often limited or even lacking in the lives of persons with complex mental health needs [30]. To fill this void, the ward considers it one of its core tasks to actively create enabling environments in the community for its patients, operationalized through the work of the ‘road builders’. Described by Kal as *quarter making* (translated from the Dutch ‘kwartiermaken’), their work does not take place within the ward, but explicitly aims to open up welcoming spaces in society in which patients can participate and belong as inclusive citizens [28]. In other words, our findings show how, in the daily practice of the ward, interdependence and connectedness are the driving forces that power other processes of installing hope, establishing a sense of identity, finding meaning in life and empowerment [7]. Related to the above reflections, Quirk et al. refer to the concept of institutional *permeability* to describe the extent to which psychiatric (inpatient) settings are interacting with the outside world [31]. The findings of our study show that the ward’s high degree of permeability is an indispensable facilitator in the patients’ recovery journeys.

Whilst the daily practice of the ward shows a lot of common ground with the recovery framework, it also remains interwoven with the more traditional institutional culture of the hospital in which it is located. In that respect, two critical side notes need to be made. First, under impulse of Goffman’s *Asylums* and the subsequent deinstitutionalization wave, the image of psychiatry as totalitarian and hermetically sealed institutions was gradually rejected and replaced by a focus on community-oriented support [31, 32]. However, the negative connotation of these outdated institutions as asylums should not be confused with the basic asylum function of psychiatry, i.e. *“the provision of safety and security for individual patients needing refuge”* ([33], p. 976). In our study, the importance of this asylum function clearly came to the fore: patients experience the ward as an inviolable place, both in terms of space (cf. *feeling safe*) and time (cf. *the ward as a pit stop*), where they find rest and feel contained. However, in today’s recovery-oriented and community-based mental health care policy, in which the focus lies on supporting people with mental health problems to live independently in the community, the provision of this asylum function risks being overlooked or even ignored [34].

Second, although the ward aims to help patients gain perspective on a meaningful life in the community, the results also showed that it can be particularly challenging to do so with patients who have lived at the ward for many years, as they experience the hospital as their home. One participant attributes this difficulty to the fact that these patients suffer from the *“hospitalization syndrome”*, i.e. the inability to imagine a life outside the hospital walls. Alternatively, however, this (apparent) lack of imagination could also be explained as a side-effect of the ward’s former institutional vision that some patients should be given life-long protection from a threatening outside world. Today, the ward has shifted to a recovery-oriented vision in which patients are seen as active agents of their lives and are encouraged to (re)connect with social roles and aspirations outside the hospital. A potential danger of this activating and individualized approach is that difficulties in orienting patients towards a valuable community life are translated as a matter of reluctance or inability on the patients’ side, e.g. by saying that they suffer from a *“hospitalization syndrome”*, rather than considered the shared responsibility of different parties (e.g. hospital, patients, actors in the community). In other words, when the psychiatric institute represses its own historical evolution, it risks slipping into a neoliberal mindset in which patients are exclusively seen as individuals, self-managing and self-responsible for the success or failure of their therapeutic trajectory [35, 36].

The above-described findings of this study do not only provide us with insight into the roles of inpatient settings in mental health recovery, but also point to important insights regarding the support and treatment provision for persons with complex mental health needs. First of all, the results resonate well with existing evidence that support should be organized from a person-centered and holistic perspective (cf. ‘a tailor-made approach’), in which persons with complex mental health needs themselves hold the compass that directs their support trajectories [37, 38]. This, amongst other things, requires a specific attitude from mental health care professionals who should take on the role of travel companions that engage in an on-going dialogue with service users about their needs, aspirations and meaning of recovery. In that respect, relational continuity of support, i.e. having long-standing therapeutic contacts and relationships provide service users with a sense of coherence throughout their unpredictable recovery trajectories [39]. For example, in that sense, the ward’s function as a ‘pit stop’ is of great importance in providing such continuity. Additionally, the findings of this study strongly show that, to be able to provide person-centered support that takes shape in dialogue with service users, organizations need to be granted space to improvise and to color outside the lines of more formal and traditional support options (cf. ‘a continuous (re)negotiation’). However, the need for this improvisational space is often at odds with today’s mental health care policy logic that is characterized by a desire for effectiveness and measurable successes [17, 40]. Consequently, short-term treatment programs in which service users’ progress can easily be evaluated risk being favored over settings (such as the ward under study) that apply a long-term vision on recovery and leave room for ups and downs and the impact of challenging living circumstances.

### Future directions

Up until today, research into recovery-oriented practice has largely focused on outpatient and community-based initiatives [2]. Although our study is atypical in this respect, as it focuses on a residential psychiatric ward, it illustrates how inpatient settings play valuable roles in the recovery processes of persons with complex mental health needs. Remarkably, our study shows striking similarities with the results of another study that focused on the daily practice of an alternative community-based meeting place. Whilst this setting can be situated at the other end of the mental health care continuum to an inpatient ward, it engages with a similar group of persons with complex mental health needs [37]. Like the inpatient ward, the meeting place is experienced as a safe haven where visitors (as clients are called) feel welcome and accepted. This is done by having a horizontal structure and by organizing activities on a voluntary basis (e.g. visitors can come and go as they wish). In that respect, the meeting place functions as *a place to be*, or even as *the* place to be, as visitors experience the place as their ‘second home’. These characteristics tally well with the asylum function of the ward in the current study, in which aspects such as feeling safe and feeling ‘at home’ also stand out. Besides a safe haven, the meeting place also functions a lively hub in which all kinds of (artistic, creative, sports) activities are organized, all rooted in the personal interests of the visitors. Through these activities, visitors are encouraged to find a medium to engage in dialogue with others and to establish a personal sense of identity (e.g. artist, musician, cook, yogi) that moves away from other stigmatizing identities. Because these activities often take place in the community (especially through the performing arts), they help visitors become ‘visible’ citizens in society. In that sense, the meeting place functions as *a place to be me*. In this function, several commonalities can be found with the ward as a *particularizing* and *transitional space*, such as the function of ‘quarter making’ (cf. supra), the focus on inclusive citizenship and the importance of creating space for patients to (re)connect with their identity (e.g. by not having an obligatory set of activities).

Despite their diverse contexts, the similarities between these studies show how recovery-oriented support is not the exclusive terrain of community-oriented initiatives. Moreover, the daily practice of the ward also shows that a recovery-oriented approach is not irreconcilable with an institutional approach to support. It is precisely in the entanglement of different approaches and in the complementarity and diversity of different support settings that persons with complex mental health needs find the resources for their recovery. Therefore, we propose to think about recovery-oriented systems of support in terms of differentiated landscapes in which persons with mental health needs can circulate between different enabling places, tailored to the ebb and flow of their recovery process [41–43]. As these enabling places can take various shapes (e.g. residential/community-based, professional/informal), future research is necessary to further explore the diversity of such landscapes of support and to gain understanding of the ways in which persons with complex mental health needs navigate them. At the same time, we are aware of the fact that mental health care is an extremely complex domain which is continuously under the influence of different organizational, systemic, political, economic, social and societal developments. The present study primarily aimed to gain insight into the daily practice of the ward from an insider perspective, i.e. by focusing on the personal perspectives of the patients and staff members who experience it first-hand. Although experience-based research provides us with an indispensable view on the roles of (inpatient) mental health care settings, future research is necessary to unravel and untangle the impact of larger-scale processes and dynamics on the recovery-oriented capacities of mental health care landscapes.

## Data Availability

The datasets used and analyzed during the current study are available from the corresponding author on reasonable request.
